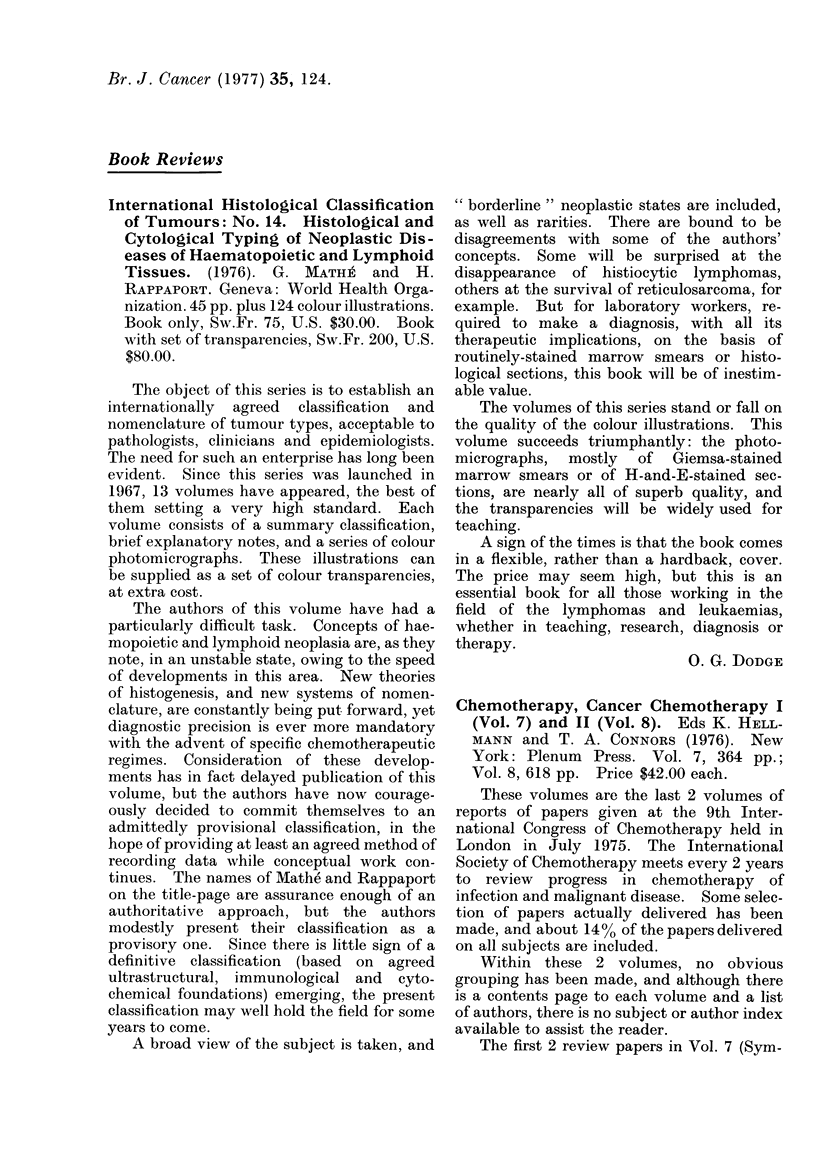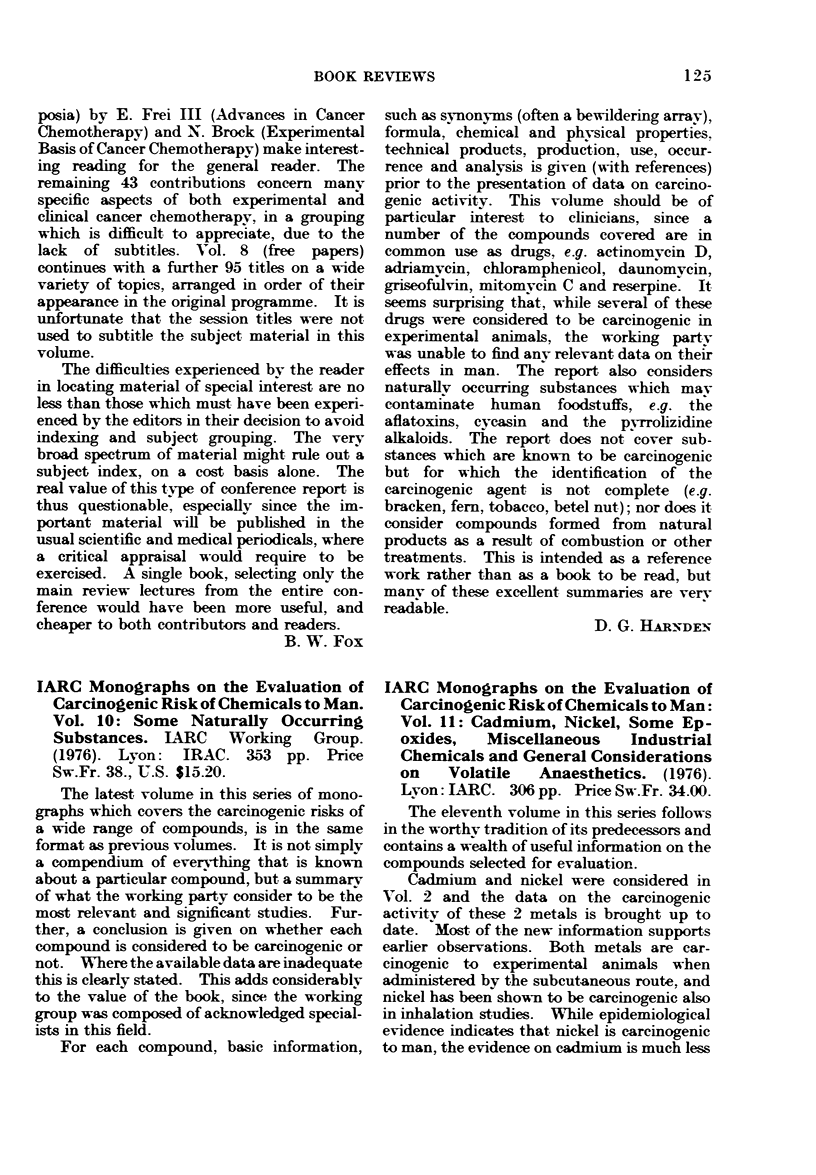# Chemotherapy, Cancer Chemotherapy I (Vol. 7) and II (Vol. 8)

**Published:** 1977-01

**Authors:** B. W. Fox


**Chemotherapy, Cancer Chemotherapy I (Vol. 7) and II (Vol. 8).** Eds K. Hellmann and T. A. Connors (1976). New York: Plenum Press. Vol. 7, 364 pp.; Vol. 8, 618 pp. Price $42.00 each.

These volumes are the last 2 volumes of reports of papers given at the 9th International Congress of Chemotherapy held in London in July 1975. The International Society of Chemotherapy meets every 2 years to review progress in chemotherapy of infection and malignant disease. Some selection of papers actually delivered has been made, and about 14% of the papers delivered on all subjects are included.

Within these 2 volumes, no obvious grouping has been made, and although there is a contents page to each volume and a list of authors, there is no subject or author index available to assist the reader.

The first 2 review papers in Vol. 7 (Symposia) by E. Frei III (Advances in Cancer Chemotherapy) and N. Brock (Experimental Basis of Cancer Chemotherapy) make interesting reading for the general reader. The remaining 43 contributions concern many specific aspects of both experimental and clinical cancer chemotherapy, in a grouping which is difficult to appreciate, due to the lack of subtitles. Vol. 8 (free papers) continues with a further 95 titles on a wide variety of topics, arranged in order of their appearance in the original programme. It is unfortunate that the session titles were not used to subtitle the subject material in this volume.

The difficulties experienced by the reader in locating material of special interest are no less than those which must have been experienced by the editors in their decision to avoid indexing and subject grouping. The very broad spectrum of material might rule out a subject index, on a cost basis alone. The real value of this type of conference report is thus questionable, especially since the important material will be published in the usual scientific and medical periodicals, where a critical appraisal would require to be exercised. A single book, selecting only the main review lectures from the entire conference would have been more useful, and cheaper to both contributors and readers.

B. W. Fox